# Impact of relative dose intensity (RDI) in CHOP combined with rituximab (R-CHOP) on survival in diffuse large B-cell lymphoma

**DOI:** 10.1186/1756-9966-28-116

**Published:** 2009-08-19

**Authors:** Yoshiki Terada, Hirohisa Nakamae, Ran Aimoto, Hiroshi Kanashima, Erina Sakamoto, Mizuki Aimoto, Eri Inoue, Hideo Koh, Takahiko Nakane, Yasunobu Takeoka, Masahiko Ohsawa, Ki-Ryang Koh, Takahisa Yamane, Yoshitaka Nakao, Kensuke Ohta, Atsuko Mugitani, Hirofumi Teshima, Masayuki Hino

**Affiliations:** 1 Hematology, Graduate School of Medicine, Osaka City University, Osaka, Japan; 2 Department of Hematology, Osaka City General Hospital, Osaka, Japan; 3 Department of Pathology, Graduate School of Medicine, Osaka City University, Osaka, Japan; 4 Department of Hematology, Wakakoukai Hospital, Osaka, Japan; 5 Department of Hematology, Saiseikai Nakatsu Hospital, Osaka, Japan; 6 Department of Hematology, Seichokai Fuchu Hospital, Osaka, Japan

## Abstract

**Background:**

Recently, maintaining higher relative dose intensity (RDI) of chemotherapeutic drugs has become a widespread practice in an attempt to achieve better outcomes in the treatment of aggressive lymphoma. The addition of rituximab to chemotherapy regimens has significantly improved outcome in diffuse large B-cell lymphoma (DLBL). However, it is unknown if higher RDI in chemotherapy when combined with rituximab leads to a better outcome in aggressive B-cell lymphoma.

**Methods:**

We retrospectively evaluated the impact of the RDI of initial chemotherapy (consisting of cyclophosphamide, doxorubicin, vincristine and prednisolone with rituximab (R-CHOP) on outcome in 100 newly diagnosed DLBL patients.

**Results:**

A multivariate Cox regression model showed that RDI trended towards a significant association with mortality [hazard ratio per 0.1 of RDI = 0.8; 95% confidence interval 0.6–1.0; *P *= 0.08]. Additionally, on multivariate logistic analysis, advanced age was a significant factor for reduced RDI.

**Conclusion:**

Our data suggest that in DLBL patients, mortality was affected by RDI of R-CHOP as the initial treatment, and the retention of a high RDI could therefore be crucial.

## Background

Aggressive lymphoma is known to be a highly chemosensitive disease. Therefore, over the past few decades, constant attempts have been made to develop various types of combination chemotherapy including first generation combination chemotherapy with cyclophosphamide, doxorubicin, vincristine and prednisone (CHOP) [1]. However, particularly in patients with aggressive lymphoma in the higher International Prognostic Index (IPI) risk group, satisfactory outcomes have not been achieved, with a five-year survival of less than 50% [2].

Several retrospective studies demonstrated that the relative dose intensity (RDI) of combination chemotherapy significantly influences survival in aggressive lymphoma [3-7].

Moreover, rituximab, a chimeric monoclonal anti-CD20 antibody combined with CHOP chemotherapy (R-CHOP) has improved outcome in patients with diffuse large B-cell lymphoma (DLBL) [8,9]. Rituximab has direct, complement-dependent and antibody-dependent cellular cytotoxicity against B-cells. The drug also sensitizes B-lymphoma cells to chemotherapy [10]. Therefore, a combined approach with rituximab plus CHOP could conceivably modify the effects of RDI. However, there is no evidence that even in combination chemotherapy with rituximab that higher RDI improves the outcome for aggressive B-cell type lymphoma. Hence, in our study, we retrospectively analyzed the impact of the RDI of chemotherapy with R-CHOP as an initial treatment on the survival of patients with DLBL, and furthermore, we determined the factors influencing RDI.

## Methods

### Eligibility

Patients were eligible if they had newly diagnosed DLBL according to the World Health Organization classification or the Revised European-American Lymphoma classification [11,12]. As initial chemotherapy, they received R-CHOP with more than three consecutive courses between December 2003 and February 2008 at five institutions, Osaka City University Hospital, Osaka City General Hospital, Seichokai Fuchu Hospital, Saiseikai Nakatu Hospital and Wakakoukai Hospital. One hundred patients who had complete records of drug dose, time intervals, and prophylactic G-CSF use were deemed eligible for this study. Patients were excluded if they had T-cell lymphoma or prior radiotherapy before CHOP. Clinical data and follow-up information were obtained by reviewing the patients' medical records. All patients provided written informed consent for their treatment.

### Patient Characteristics

We analyzed 100 newly diagnosed DLBL patients treated with initial R-CHOP chemotherapy. The clinical characteristics of all the patients are shown in Table 1. Median age of the patients was 60 years. Of the 100 patients, 45 were 61 years or older. Sixty-two patients had advanced-stage (stage III, IV) disease, and 23 patients had poor performance status (PS). In 52 patients, lactate dehydrogenase level (LDH) was high (over the upper limit of normal). Thirty-two patients had two or more extranodal disease sites. Forty-two patients were in the higher IPI risk group (high or high-intermediate risk group). In 26 patients, serum albumin levels were < 3.5 g/dl. The median number of CHOP courses was 6 (range, 3–8). The median number of R-CHOP cycles for patients with localized disease was 6 (range, 3–8), and there was no significant difference in the number of cycles between patients with localized disease and those with advanced disease.

**Table 1:** Patient characteristics

	n. (%)
Total number of patients	100
Age	
< 61	55 (55)
≥ 61	45 (45)
Clinical Stage	
I, II	38 (38)
III, IV	62 (62)
Performance status	
0–1	77 (77)
2–4	23 (23)
LDH	
N≥	52 (52)
N <	48 (48)
Extranodal lesion	
0–1	68 (68)
2–4	32 (32)
IPI	
Low/low-intermediate	58 (58)
High/high-intermediate	42 (42)
Albumin	
< 3.5 g/dl	26 (26)
≥3.5 g/dl	74 (74)
Prophylactic G-CSF	
yes	62 (62)
no	38 (38)

N: normal range; IPI: international prognostic index; G-CSF: granulocyte colony-stimulating factor

### Chemotherapy Regimen

The CHOP chemotherapy consisted of cyclophosphamide (750 mg/m^2 ^given intravenously on Day 1), doxorubicin (50 mg/m^2 ^given intravenously on Day 1), vincristine (1.4 mg/m^2 ^(maximum 2 mg/body), given intravenously on Day 1) and prednisolone (100 mg/day, given orally on Day 1 to 5) [13]. The treatment course was repeated every three weeks, unless peripheral leukocyte or platelet counts became too low to administer the next cycle. A time limit for peripheral blood count recovery before administration of the next cycle of chemotherapy was not adopted. In patients who experienced severe neutropenia, thrombocytopenia and/or infections, or febrile neutropenia during cycles, the doses of cyclophosphamide, doxorubicin and vincristine in the subsequent cycle were reduced at the discretion of clinical physicians. Moreover, the dose of vincristine was also reduced depending on the occurrence and degree of neurologic toxicity. Rituximab was administered at a dose of 375 mg/m^2 ^per cycle for up to 8 cycles concurrently with CHOP, as long as the disease responded to the treatment. Seven patients received involved-field radiation therapy of 30–40 Gy.

G-CSF was given if patients experienced neutropenia with an absolute neutrophil count of less than 500/μl during cycles. Prophylactic G-CSF was administered at the physician's discretion to prevent the development of neutropenia in 62 patients who had experienced infections associated with neutropenia in the prior cycle [14]. In these patients, the median number of CHOP cycles with prophylactic G-CSF was 3 (range, 1–6).

### Calculation of Dose Intensity (DI)

The DI of each agent was calculated by dividing the total received dose of the agent by the number of weeks of treatment [3]. The relative total dose intensity (RTDI) of each agent was calculated by expressing the total delivered dose of agent per unit time (week) as a percentage of the target dose. The averaged RDI (ARDI) was calculated by expressing the average delivered dose of the chemotherapy regimen per unit time (week) as a percentage of the target dose. In this study, the ARDI was calculated by averaging the RTDIs of cyclophosphamide and doxorubicin in all the chemotherapy courses, and hereinafter the ARDI of R-CHOP is simply referred to as the "RDI."

### Statistical Methods

Overall survival (OS) was calculated from the initiation of R-CHOP chemotherapy to the time of death or to the time of the last follow-up. Progression free survival (PFS) was calculated from the initiation of R-CHOP chemotherapy to the time of relapse, progression, death or the last follow-up. Both OS and PFS were calculated using the Kaplan-Meier method. Survival curves of the different groups were compared using the log-rank test. Univariate and multivariate Cox proportional hazard regression analyses were used to assess the effects of the pretreatment prognostic factors on overall survival [15]. Multiple logistic analysis was applied to identify factors influencing RDI. *P *values less than 0.05 were considered to be statistically significant, and all tests were two-tailed. All analyses were performed using SPSS version 15.0 J (SPSS, Chicago, IL).

## Results

### RDI

In all patients, the calculated medians of the RTDI of doxorubicin and cyclophosphamide were 88.8% and 88.6%, respectively and the median RDI for all cycles of R-CHOP given was 87.9%.

### Survival Analysis

**Figure 1:**
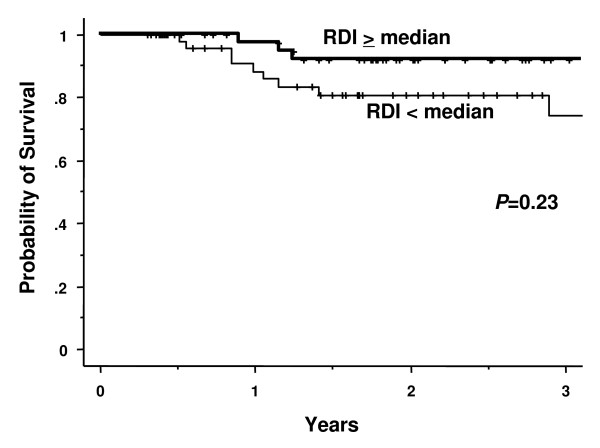
**Overall survival curves of the higher RDI (≥ median) and the lower RDI (< median) group**. RDI: relative dose intensity (RDI) of R-CHOP chemotherapy.

We registered 14 deaths. With a median follow-up of 21.2 months, the three-year OS in all cases, in the group with a higher RDI (above the median) and in the group with a lower RDI (below the median) was 81.6%, 92.1% and 74.2%, respectively (Figure 1). The three-year PFS in all cases, in the group with a higher RDI (above the median) and in the group with a lower RDI (below the median) was 56.3%, 58.7% and 54.0%, respectively.

In the univariate analysis to identify prognostic factors for OS, RDI and IPI were significant factors influencing OS. In a multivariate analysis, RDI tended to be a significant risk factor for mortality [hazard ratio (HR) per 0.1 of RDI = 0.8; 95% confidence interval (CI) 0.6–1.0; *P *= 0.08] (Table 2).

**Table 2:** Prognostic factors for overall survival

	Univariate model		Multivariate model	
Factor	HR (95%CI)	*P *value	HR (95%CI)	*P *value

Age (61 ≤)	2.2 (0.8–6.7)	0.15	-	-
Sex (male)	2.6 (0.7–9.3)	0.14	-	-
Stage III, IV	7.6 (1.0–5.8)	0.15	-	-
Extranodal site (2 ≤)	1.7 (0.6–4.8)	0.35	-	-
LDH (> upper normal limit)	1.8 (0.5–5.8)	0.34	-	-
Performance status (2–4)	2.8 (1.0–8.1)	0.05	-	-
RDI (CPA+DOX) per 0.1	0.7 (0.6–0.9)	0.02*	0.8 (0.6–1.0)	0.08
IPI (high/high intermediate)	4.7 (1.3–17)	0.02*	3.8 (1.0–14)	0.05
Albumin (3.5 mg/dl ≤)	0.7 (0.4–1.2)	0.20	-	-
Prophylactic G-CSF	1.6 (0.5–4.9)	0.44	-	-

HR: hazard ratio; CI: confidence interval; RDI: relative dose intensity; CPA: cyclophosphamide; DOX: doxorubicin; G-CSF: granulocyte colony-stimulating factor

### Factors Influencing RDI

The univariate analyses identified advanced age and higher IPI score as risk factors for reduced RDI. In the multivariate logistic analysis of all these factors, only older age remained as a factor that retained persistent statistical significance [odds ratio (OR) = 0.4; 95% CI 0.2–0.8; *P *= 0.02]. (Table 3).

**Table 3:** Factors influencing RDI (above the Median): Univariate and Multivariate analysis

	Univariate model		Multivariate model 1		Multivariate model 2	
Factor	OR (95%CI)	*P *value	OR (95%CI)	*P *value	OR (95%CI)	*P *value

Age (61 ≤)	0.3(0.2–0.8)	0.0099*	0.4 (0.2–0.8)	0.06	0.4 (0.2–0.8)	0.02*
Sex (male)	1.3 (0.6–2.9)	0.54	-	-	-	-
Stage III, IV	0.8 (0.4–1.9)	0.68	-	-	-	-
Extranodal site (2 ≤)	1.0 (0.4–2.3)	1.00	-	-	-	-
LDH (> upper normal limit)	0.5 (0.2–1.2)	0.11	-	-	0.6 (0.3–1.4)	0.24
Performance status (2–4)	0.6 (0.2–1.5)	0.24	-	-	-	-
IPI (high/high intermediate)	0.4 (0.2–1.0)	0.04*	0.6 (0.3–1.6)	0.33	-	-
Alb (3.5 mg/dl >)	0.8 (0.5–1.4)	0.50	-	-	-	-
Prophylactic G-CSF +	1.7 (0.7–3.8)	0.22	-	-	-	-

IPI: international prognostic index. G-CSF: granulocyte colony-stimulating factor

## Discussion

In DLBL patients, our data demonstrated that a high RDI of CHOP trended towards a significant association with better survival, even when the CHOP was combined with rituximab. Only advanced age was identified as a risk factor for reduced RDI.

There are several previous studies of the relationship between the RDI of chemotherapy and survival in aggressive lymphoma. A high RDI of doxorubicin in CHOP, M-BACOD, or MACOP-B chemotherapy [4], a high RDI of each drug (cyclophosphamide, doxorubicin or vincristine) and a high averaged RDI of these three drugs in CHOP for diffuse large cell lymphoma (DLCL) reportedly had a significant, positive impact on survival [5]. In addition, in ACVB chemotherapy for aggressive lymphoma, the averaged RDI of doxorubicin and cyclophosphamide was strongly associated with survival [6]. Furthermore, it was reported that in elderly patients with DLCL who received a higher dose of doxorubicin, the outcomes were comparable to those of young patients [16]. In a recent study, a Belgian group also reported that maintaining a high RDI resulted in a favorable outcome in CHOP chemotherapy for DLBL patients [7].

The addition of rituximab to CHOP or other chemotherapy regimens has reportedly led to a significant improvement in the prognosis of DLBL patients. Interestingly, it was suggested from preclinical models that rituximab chemosensitized drug-resistant B-lymphoma cells through down-regulation of anti-apoptotic factors and endogenous IL-10 expression [17,18], suggesting that rituximab is likely to have a significant therapeutic effect by augmenting the effect of anticancer agents in CHOP in a synergistic fashion and thereby compensating for a low CHOP RDI. However, from our results, it was clear that maintaining a high RDI remained crucial in the use of R-CHOP for DLBL patients, in a similar fashion to CHOP alone.

We identified advanced age as the only factor that reduced RDI. A nationwide study of RDI in CHOP-like chemotherapies in patients with non-Hodgkin's lymphoma (NHL) in the United States also showed that older age was a risk factors for reduced RDI, in addition to lack of use of prophylactic colony stimulating factor (CSF), advanced disease stage, poor PS and a lower serum albumin level [19]. Moreover, the study indicated that prophylactic CSF use is important in maintaining a high RDI, particularly in elderly patients. The American Society of Clinical Oncology update guideline for the use of CSF, also recommends use of prophylactic CSF during curative and intensive chemotherapy for elderly patients with DLCL, to reduce the incidence of febrile neutropenia and infections [14]. In addition, according to the European Organization for Research and Treatment of Cancer guideline on the use of G-CSF, when dose-dense or dose-intense chemotherapy has a survival benefit, prophylactic G-CSF use is recommended, especially in elderly patients [20]. Indeed, in a prospective study on prediction of febrile neutropenia in the first cycle of chemotherapy for NHL, elderly patients were identified as candidates for primary CSF prophylaxis [21]. Taking into account these reports as well as our results, prophylactic use of CSF could be recommend, at least in elderly patients with DLBL who are scheduled to receive R-CHOP chemotherapy in order to maintain RDI.

As our study was a retrospective cohort study with a small study population and/or short median follow-up periods, it was inevitable that treatment bias due to physician discretion in making treatment decisions would arise. Therefore, prospective randomized trials will be required to confirm the value of maintaining a high RDI. For instance, an alternative strategy to intensify RDI by shortening the intervals between cycles of chemotherapy, such as bi-weekly CHOP, may be promising [22]. Indeed, Groupe d'Etudes de Lymphomes de L'Adulte (GELA) is now conducting a phase III prospective randomized trial to assess the difference between eight cycles of bi-weekly R-CHOP and three-weekly R-CHOP. The results of this prospective randomized trial are awaited.

## Conclusion

In DLBL patients, mortality was affected by the RDI of R-CHOP as the initial treatment and the retention of high RDI could be crucial, especially in elderly patients. To optimize the RDI of conventional chemotherapy in order to achieve better outcomes for patients with DLBL, further investigation of RDI will be required.

## Competing interests

The authors declare that they have no competing interests.

## Authors' contributions

All the authors contributed as mentioned. YT and HN conceived of the study and drafted the manuscript. RA obtained clinical data and follow-up information by reviewing the patients' medical records. MO reviewed the pathological specimens in this study. HK, ES, MA, EI, HK, TN, YT, MO, KK, TY, YN, KO, AM, and HT participated in designing the study and helped to write the paper. MH supervised the entire study. All authors have read and approved the final manuscript.
